# A Case of Subnormal Temperature1Read before the Virginia State Veterinary Medical Association, June 24, 1896.

**Published:** 1896-09

**Authors:** W. H. Harbaugh


### REPORTS OF CASES.

### REPORT OF A CASE OF SUBNORMAL TEMPERATURE.[1]

BY W. H. HARBAUGH, V.S.

OCTOBER 4th, 10 P.M., chestnut gelding brought to hospital; symptoms of aggravated heaves.

Owner had noticed the abnormal breathing and cough for the past three weeks, but never saw the horse in such an alarming condition as he found him this night. Owner stated that the food was good so far as he was able to judge.

I attributed the condition to either an over-feed, or feed of bad quality, and administered a bolus composed of aloes, sodium bicarbonate, and creolin. I also gave on the tongue a dose of fluid extract of cannabis indica to allay the cough, which it did in a short time. Owner said that the horse had been worked to buggy very little each day for some time past. Horse taken home.

October 5th. Horse brought back about 8 A.M., still heaving very much at flanks, and with a new complication in the form of violent spasms of the diaphragm. On taking the temperature I was surprised to find it 95.4° F.

Thinking there might be a mistake, I took it again with the same result. I ordered alcoholic stimulants, and gave a dose immediately. Horse taken home again.

October 6th. Horse brought back at 9 A.M. He is bright and cheerful, showing no signs whatever of any ailment. Temperature with same thermometer, 100.2° F. Prescribed a course of liquor acidi arseniosi, and gave proper directions in regard to feed and water. The animal returned to his usual work, and is doing well. The only explanation I can offer for this remarkable subnormal temperature is the severe shock to the nervous system by the derangement of the digestive functions. Physical

[1] Read before the Virginia State Veterinary Medical Association, June 24, 1896.

examination failed to discover any organic disease of heart or lungs. An extended experience with creolin in an aloetic bolus both before and since convinces me that it was no factor in reducing the temperature.

